# Influenza virus and endothelial cells: a species specific relationship

**DOI:** 10.3389/fmicb.2014.00653

**Published:** 2014-12-02

**Authors:** Kirsty R. Short, Edwin J. B. Veldhuis Kroeze, Leslie A. Reperant, Mathilde Richard, Thijs Kuiken

**Affiliations:** ^1^Department of Viroscience, Erasmus Medical CentreRotterdam, Netherlands; ^2^School of Biomedical Sciences, University of QueenslandBrisbane, QLD, Australia

**Keywords:** influenza virus, endothelial cells, highly pathogenic avian influenza, zoonotic infection, poultry

## Abstract

Influenza A virus (IAV) infection is an important cause of respiratory disease in humans. The original reservoirs of IAV are wild waterfowl and shorebirds, where virus infection causes limited, if any, disease. Both in humans and in wild waterbirds, epithelial cells are the main target of infection. However, influenza virus can spread from wild bird species to terrestrial poultry. Here, the virus can evolve into highly pathogenic avian influenza (HPAI). Part of this evolution involves increased viral tropism for endothelial cells. HPAI virus infections not only cause severe disease in chickens and other terrestrial poultry species but can also spread to humans and back to wild bird populations. Here, we review the role of the endothelium in the pathogenesis of influenza virus infection in wild birds, terrestrial poultry and humans with a particular focus on HPAI viruses. We demonstrate that whilst the endothelium is an important target of virus infection in terrestrial poultry and some wild bird species, in humans the endothelium is more important in controlling the local inflammatory milieu. Thus, the endothelium plays an important, but species-specific, role in the pathogenesis of influenza virus infection.

## Introduction

Influenza A virus (IAV) is a negative-sense RNA virus of the Family *Orthomyxoviridae*. IAVs can be classified into different subtypes based on antigenic differences in the two surface glycoproteins of the virus, the hemagglutinin (HA) and neuraminidase (NA). Each year, antigenic changes (or “drift”) in the HA and NA result in a seasonal outbreak of IAV in the human population. However, when there is a dramatic change in the HA and/or NA, often originating from the avian reservoir, global pandemics can result. In the last 100 years there have been four IAV pandemics in the human population: the 1918 H1N1 pandemic, the 1957 H2N2 pandemic, the 1968 H3N2 pandemic and the 2009 H1N1 pandemic. These pandemics have all caused significant morbidity and mortality. Indeed, the 1918 H1N1 virus was so severe that life expectancy in the U.S.A. dropped by 11.8 years from 1917 to 1918 (Noymer and Garenne, 2000). The constant threat of a future IAV pandemic highlights the need to study and understand IAV pathogenesis not only in humans, but also in the virus' natural avian reservoirs. The original reservoirs of IAV are wild waterfowl (order Anseriformes—which includes geese, ducks, and swans) and shorebirds (order Charadriiformes—which includes gulls and waders). Sixteen different HA subtypes and 9 different NA subtypes of IAV have been recorded in wild waterfowl. A subset of these different IAV strains can then spread from wild bird populations to terrestrial poultry (order Galliformes). Here, IAV can cause a mild or subclinical infection of the respiratory and/or gastrointestinal tract, and is thus referred to as a low pathogenic avian influenza (LPAI) virus. LPAI viruses of the H5 and H7 subtypes can subsequently evolve in poultry to become highly pathogenic avian influenza (HPAI) viruses, which typically cause a fatal and systemic infection. LPAI and HPAI viruses can cause respiratory infection in humans, with HPAI viruses occasionally reported in extra-respiratory organs.

The pathogenesis of IAV infection differs markedly between wild waterbirds, terrestrial poultry and humans. In all three host groups, endothelial cells play a key role in disease pathogenesis—either as the primary cellular target of viral infection or as orchestrators of the anti-viral immune response. Here, we review the currently available literature on the role of the endothelium in the pathogenesis of IAV (in particular HPAI H5N1 viruses) in terrestrial poultry, wild birds and humans. Specifically, we will compare the ability of IAV to infect and/or “activate” the endothelium across these different host groups.

## Terrestrial poultry (order galliformes)

**Figure 1:**
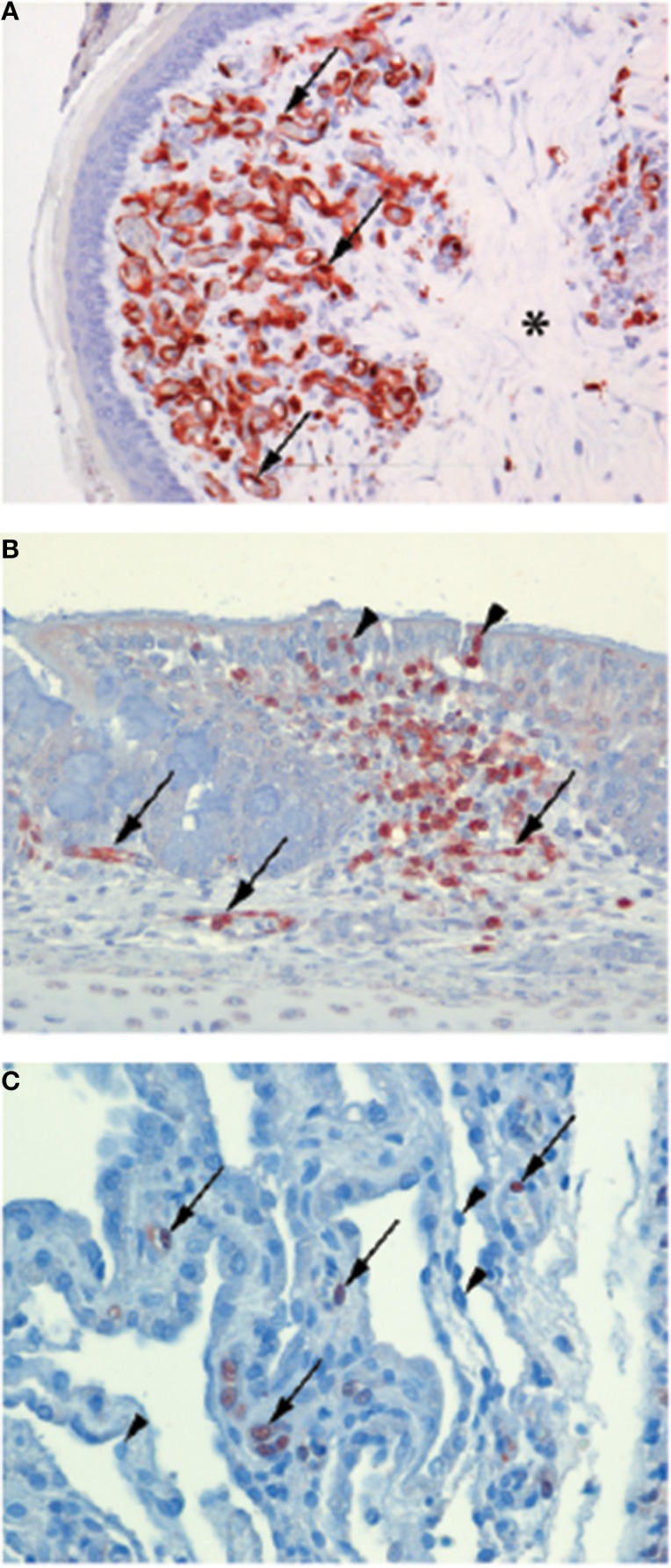
**Endotheliotropism and epitheliotropism of IAVs in chickens**. Virus distribution in: **(A)** endothelial cells of the wattle of a chicken naturally infected with HPAI H7N7 virus. Brown-reddish staining antigen indicative of viral replication is present in many endothelial cell nuclei (arrows) and cytoplasm lining the small blood vessels. The dermis is expanded by edema (asterisk). (original magnification 200×, in van Riel et al. (2009) **(B)** Epithelial cell nuclei (arrowheads) and in endothelial cells (arrows) of the nasal mucosa of a chicken 24 h after experimental intranasal infection with 10^5^ TCID_50_ of HPAI H5N1 virus A/Indonesia/05/2005, illustrating both epitheliotropism and endotheliotropism of the virus (original magnification 200×). **(C)** Endothelial cell nuclei (arrows) of the lung interstitium 24 h after infection of the same chicken as in **(B)**, the alveolar lining epithelial cells nuclei (arrowheads) do not stain positive for viral antigen (original magnification 400×). (Immunohistochemistry (IHC) for IAV-nucleoprotein (NP) with hematoxylin counterstain).

Typically, terrestrial poultry infected with LPAI viruses display limited clinical signs and with no evidence of endothelial cell infection (rather the virus preferentially infects epithelial cells of the respiratory tract) (Hooper and Selleck, 2003). However, viruses of the H5 and H7 subtype can evolve in terrestrial poultry to become HPAI. This evolution typically occurs via the addition of a multi-basic cleavage site in the viral HA. This then allows the HA to be cleaved (a prerequisite for viral infection) by the ubiquitously present furin family of enzymes. In contrast, LPAI can only be cleaved by trypsin like enzymes that are present within the respiratory and digestive tract. Upon evolution to HPAI, the cell tropism of IAV changes dramatically. Studies on naturally or experimentally infected chickens show that HPAI can infect the endothelium in a variety of different organs including, but not limited to, the lung, heart, brain, and spleen (Brown et al., 1992; Suarez et al., 1998; Ito et al., 2002; Jones and Swayne, 2004; Nakatani et al., 2005; Muramoto et al., 2006; Swayne, 2007; Nakamura et al., 2008; van Riel et al., 2009; Wibawa et al., 2013) (see Figure 1). This endothelial cell tropism can be so striking that in chickens infected with H5N1, IAV antigen is more prevalent in the endothelial cells of the respiratory and intestinal tract than in the epithelial cells of the same tissues (Wibawa et al., 2013). Similarly, in Galliformes other than chickens viral antigens are predominant in the vascular and capillary endothelial cells of various tissues including the lung, liver, brain, skeletal muscle, pancreas, heart, kidney, spleen, and bursa (Perkins and Swayne, 2001, 2003; Lee et al., 2005; Bertran et al., 2011, 2013). The endothelial tropism of HPAI viruses is determined, at least in part, by the presence of the multi-basic cleavage site in the HA, as the removal of these basic amino acid residues reduces endothelial tropism (Schat et al., 2012). The polarity of virus budding may also contribute this distinct pattern of viral infection (Feldmann et al., 2000).

The endothelial tropism of HPAI viruses in chickens and other Galliformes has important pathological ramifications. Firstly, IAV infection of chicken endothelial cells is associated with the apoptotic death of these cells (Ito et al., 2002). The loss of endothelial cells is likely to contribute to the oedema and hemorrhaging observed in the wattle, comb, lungs and legs of chickens infected with HPAI viruses (Perkins and Swayne, 2002a; van Riel et al., 2009). A loss of endothelial cells can also detrimentally affect blood coagulation (Muramoto et al., 2006; Swayne, 2007). Damage to endothelial cells activates the extrinsic coagulation cascade and facilitates the microthrombosis. This can then lead to disseminated intravascular coagulation (DIC) whereby the coagulation cascade become “hyper-activated,” resulting in thrombocytopenia, wide-spread hemorrhaging and ischaemia. Consistent with a role for DIC in the pathogenesis of HPAI viruses in chickens, Muramoto and colleagues demonstrated that chickens intravenously infected with H5N1 display both microthrombosis and thrombocytopenia (Muramoto et al., 2006). Similarly, chickens infected intranasally with H5N1 display microthrombosis in the lung within 24 h post-infection. It has also been suggested that the replication of HPAI viruses in endothelial cells could disrupt the innate immune response (Suzuki et al., 2009), thermoregulation (Suzuki et al., 2009) and facilitate the systemic spread of the virus to parenchymal cells of the brain, skin, and visceral organs (Pantin-Jackwood and Swayne, 2009). Together, these features help account for the rapid and high mortality rates of HPAI in Galliformes. For example, in chickens death typically occurs within 2 days post-infection, often in the absence of visible clinical signs (Wibawa et al., 2013). Similarly, during an outbreak of a HPAI H7N1 virus in Italy, turkeys and guinea fowl (reared on litter) had a 100% mortality rate within a mere 48–72 h of becoming symptomatic (Mutinelli et al., 2003).

## Wild birds (order anseriformes and charadriiformes)

In wild birds, LPAI viruses normally present as a sub-clinical infection with little involvement of the endothelium in disease pathogenesis (Webster et al., 1992; Pantin-Jackwood and Swayne, 2009). Prior to the emergence of HPAI H5N1 viruses, there was only one recorded incidence of a HPAI strain being detected in wild birds (Becker, 1966). It was therefore assumed that HPAI strains were unlikely to transmit back to the wild bird population and cause disease following their emergence in poultry. However, since 2002 the HPAI H5N1 strain has caused infection and mortality in a variety of wild birds. Unlike LPAI viruses, H5N1 viruses in wild birds do not infect intestinal epithelial cells. Instead, the virus predominantly infects cells in the respiratory tract and other organs (see Tables 1, 2) and infection can be associated with severe necrosis and inflammation (Kwon et al., 2005; Pasick et al., 2007; Bröjer et al., 2009; Daoust et al., 2011; Wibawa et al., 2013). H5N1 viruses display, at most, limited tropism for the endothelium in wild birds (see Tables 1, 2), and it is therefore unlikely that endothelial cell infection plays a major role in disease pathogenesis. However, one notable exception to this trend is black swans (Brown et al., 2008a). Upon infection with A/whooper swan/Mongolia/244/05(H5N1), 100% (*n* = 5) of black swans succumbed to disease within 2–3 days (as seen during H5N1 infection of chickens, this was often observed in the absence of clinical signs of disease) (Brown et al., 2008a). Immunohistochemistry showed that the endothelial cells throughout the body were the primary target of IAV infection, and the presence of IAV antigen was associated with multiorgan necrosis and mild acute inflammation (Brown et al., 2008a). Although a tropism for the endothelium is observed in other species of swans, this does not appear to be as pronounced as that seen in the black swan (Ellis et al., 2004; Teifke et al., 2007; Brown et al., 2008a; Kalthoff et al., 2008; Kwon et al., 2010). For example, viral antigen was detected infrequently or not at all in the endothelial cells of whooper swans (Teifke et al., 2007; Brown et al., 2008a) and trumpeter swans (Brown et al., 2008a) following either a natural or experimental infection with H5N1. Similarly, whilst endothelial cells of mute swans were positive for IAV, and widespread hemorrhage was recorded, this was only detected in 3 out of 12 birds (Kalthoff et al., 2008). Thus, although the ability of HPAI H5N1 to infect endothelial cells contributes to the severity of the disease observed in black swans this does not necessarily hold true for other species of swans or wild birds.

**Table 1:** **Endothelial tropism of H5N1 in Anseriformes as determined by immunohistochemistry**.

**Order**	**Species**	**Infection^a^**	**Location of viral antigen**	**Virus**	**Endothelial cell infection recorded?**	**Reference**
Anseriformes	Bar-headed goose (*Anser indicus*)	N	Brain	N/A	Occasional positive endothelial cell within the gut mucosa and lung	Ellis et al., 2004
Anseriformes	Canada goose (*Branta canadensis*)	E	Brain; Heart; Intestine; Kidney; Lung; Pancreas; Proventriculus; Sciatic nerve; Skeletal muscle; Spina; Spleen; Trachea and Ventriculus	A/chicken/Vietnam/14/2005(H5N1)	Positive endothelial cells detected in sciatic nerve (1/5 geese) and respiratory tract (3/5 geese)	Pasick et al., 2007
Anseriformes	Cackling goose (*Branta hutchinsii*)	E	Adrenal gland; Brain; Liver and Pancreas	A/whooper swan/Mongolia/244/05(H5N1)		Brown et al., 2008a
Anseriformes	Canada goose (*Branta canadensis*)	N	Lung	N/A		Ellis et al., 2004
Anseriformes	Canada goose (*Branta canadensis*)	E	Trachea; Tracheal cartilage; Lung; Cerebrum; Cerebellum; Ventricles; Medulla oblongata; Spinal cord; Heart; Pancreas; Esophagus; Proventriculus; Duodenum and Ceca	A/chicken/Vietnam/14/05 (H5N1)	A few endothelial cells in scattered capillaries	Neufeld et al., 2009
Anseriformes	Domestic geese (*Anser anser domesticus*)	E	Nasal cavity; Heart; Brain; Alimentary tract; Pancreas; Liver and Spleen	A/chicken/Hong Kong/220/97(H5N1)		Perkins and Swayne, 2002a
Anseriformes	Embden geese (*Anser anser domesticus*)	E	Brain; Pancreas and Heart	A/chicken/Hong Kong/220/97 (chicken/HK) (H5N1)		Perkins and Swayne, 2003
Anseriformes	Greylag goose (*Anser anser*)	E	Brain	A/chicken/Korea/IS/06 (H5N1)		Kwon et al., 2010
Anseriformes	Hawaiian goose (*Branta sanvicensis*)	N	Lung	N/A		Ellis et al., 2004
Anseriformes	Black swan (*Cygnus atratus*)	E	Brain and Viscera	A/whooper swan/Mongolia/244/05(H5N1)	IAV antigen detected primarily in endothelial cells of blood vessels in brain and viscera	Brown et al., 2008a
Anseriformes	Coscoroba swan (*Coscoroba coscoroba*)	N	Brain and Lung	N/A		Ellis et al., 2004
Anseriformes	Mute swan (*Cygnus olor*)	E	Adrenal; Brain; Heart; Intestine; Kidney; Liver; Lung; Pancreas; Proventriculus; Spleen and Trachea	A/whooper swan/Mongolia/244/05(H5N1)		Brown et al., 2008a
Anseriformes	Mute swan (*Cygnus olor*)	E	Adrenal; Brain; Bursa; Caecum; Eye; Gonad; Heart; Kidney; Liver; Lung; Nasal cavity; Peripheral nerves; Proventriculus; Spina; Spleen and Trachea	A/Cygnus cygnus/Germany/R65/2006(H5N1)	Endothelial tropism detected in 3/12 swans in various organs including the nasal concha Blood vessel endothelial vascular endothelium in intestine	Kalthoff et al., 2008
Anseriformes	Mute swan (*Cygnus olor*)	E	Viral antigen infrequently identified in the Small and Large Intestines; Kidney; Epidermis and Pulp of feather follicles.	A/chicken/Korea/IS/06 (H5N1)	Viral antigen infrequently identified in vascular endothelium in intestine, heart and nasal cavity	Kwon et al., 2010
Anseriformes	Trumpeter swan (*Cygnus buccinators)*	E	Brain and Visceral organs	A/whooper swan/Mongolia/244/05(H5N1)		Brown et al., 2008a
Anseriformes	Whooper swan (*Cygnus cygnus*)	E	Adrenal; Brain; Heart; Intestine; Kidney; Liver; Lung; Pancreas; Proventriculus; Spleen and Trachea	A/whooper swan/Mongolia/244/05(H5N1)		Brown et al., 2008a
Anseriformes	Whooper swan (*Cygnus cygnus*)	N	Adrenal; Cerebellum; Gonad; Heart; Kidney; Liver; Lung; Pancreas; Peyer's Patches; Proventriculus; Spina; Spleen; Thyroid and Trachea	N/A	A few endothelial cells were positive within the spleen, bone marrow, Peyer's patches and lungs	Teifke et al., 2007
Anseriformes	Call ducks (*Anas platyrhyncha var domestica*)	E	Cerebrum; Cerebellum; Brain stem; Epithelium of feathers; Epithelium of beak; Pancreas; Liver; Heart and Skeletal muscle	A/chicken/Yamaguchi/7/04(H5N1)	A few positive endothelial cells were recorded. Organ not stated.	Yamamoto et al., 2007
Anseriformes	Commercial domestic ducks in Korea	N	Heart; Pancreas; Peripheral nerves and ganglia; Kidney; Skeletal myofibres; Elastic fibres of tunica media in the artery	N/A	Pulmonary endothelial cells infected	Kwon et al., 2005
Anseriformes	Domestic ducks (*Anas platyrhynchos*)	E	No viral antigen detected	A/chicken/Hong Kong/220/97(H5N1)		Perkins and Swayne, 2002a
Anseriformes	Eastern Zhejiang white geese	E	Brain, Pancreas, Lung, Spleen and Kidney	A/Bar-headed Goose/Qinghai/0510/05 (H5N1)		Zhou et al., 2006
Anseriformes	Mandarin duck (*Aix galericulata*)	E	Nasal cavity; Brain and Pancreas	A/chicken/Korea/IS/06 (H5N1)		Kwon et al., 2010
Anseriformes	Pekin ducks (*Anas platyrhyncos*)	E	No viral antigen detected	A/chicken/Hong Kong/220/97 (H5N1)		Perkins and Swayne, 2003
Anseriformes	Pekin duck (*Anas platyrhyncos*)	E	Sinus; Air sac; Ependyma; Meninges; Spleen; Bursa; Thymus; Conjunctiva; Lymphoid foci; Marrow; Periosteum; Feather sheath and follicle epidermis; Feather pulp and Myocardium Skeletal Muscle	A/duck/Sleman/BBVW-1003-34368/2007(H5N1)		Wibawa et al., 2013
Anseriformes	Pekin duck (*Anas platyrhynchos*)	E	Infraorbital sinus; Air sacs; Bursa; Thymus; Conjunctiva and Feather pulp	A/duck/Sleman/BBVW-598-32226/2007(H5N1)		Wibawa et al., 2013
Anseriformes	Pekin duck (*Anas platyrhynchos*)	E	Nasal cavity; Trachea; Lung; Heart; Brain; Adrenal gland; Enteric tract; Pancreas; Liver; Kidney; Spleen; Thymus, Skeletal muscle; Proventiculus	A/Thailand PB/6231/04(H5N1)		Pantin-Jackwood and Swayne, 2007
Anseriformes	Pekin duck (*Anas platyrhynchos*)	E	Nasal cavity; Trachea; Lung; Heart; Brain; Adrenal gland; Enteric tract; Pancreas; Liver; Kidney; Bursa; Thymus, Skeletal muscle; Proventiculus	A/Crow/Thailand/04(H5N1)		Pantin-Jackwood and Swayne, 2007
Anseriformes	Pekin duck (*Anas platyrhynchos*)	E	Nasal cavity; Trachea; Lung; Heart; Brain; Adrenal gland; Enteric tract; Pancreas; Liver; Kidney; Spleen; Bursa; Thymus, Skeletal muscle; Gizzard; Proventiculus	A/Egret/HK/757.2/02(H5N1)		Pantin-Jackwood and Swayne, 2007
Anseriformes	Ruddy shelducks (*Tadorna ferruginea*)	E	Nasal cavity, Lung; Heart; Brain; Peripheral Nerves;	A/chicken/Korea/IS/06 (H5N1)		Kwon et al., 2010
Anseriformes	Shaoxing ducks	E	pancreatic glands, brains, lungs	A/Bar-headed Goose/Qinghai/0510/05 (H5N1)		Zhou et al., 2006
Anseriformes	Tufted duck (*Aythya fuligula*)	N	Brain and neural tissue; Nasal mucosa; air vesicles and parabronchi, Liver, Pancreas; Adrenal glands; Ovarian follicular cells; Proventriculus and ventriculus	N/A	Endothelium of small vessels in the nasal mucosa positive in 2/17 ducks	Bröjer et al., 2009
Anseriformes	Tufted duck (*Aythya fuligula*)	E	Brain, Caecal tonsil; Lung; Skeletal muscle; Heart; Spleen; Liver; Airways (trachea and main bronchus)	A/turkey/Turkey/1/05(H5N1)		Londt et al., 2008
Anseriformes	Wood duck (*Aix sponsa)*^b^	E	Brain; Adrenal glands; Testicles; Kidneys; Liver, Small intestines; Heart; Skeletal muscles; Pancreas and Air sacs	A/Whooper Swan/Mongolia/244/05 (H5N1) OR A/Duck Meat/Anyang/01 (H5N1)	Endothelial cells in the brain	Brown et al., 2006

a E, Experimental; N, Natural.

b Viral antigen recorded in birds that died during the experiment.

**Table 2:** **Endothelial tropism of H5N1 in Charadriiformes as determined by immunohistochemistry**.

**Order**	**Species**	**Infection^a^**	**Location of viral antigen**	**Virus**	**Endothelial cell infection recorded?**	**Reference**
Charadriiformes	Herring gull (*Larus argentatus*)	E	Adrenal Gland; Cerebellum; Heart and Pancreas	A/whooper swan/Mongolia/244/05 (H5N1)		Brown et al., 2008b
Charadriiformes	Herring gull (*Larus argentatus*)	E	Cerebrum and Pancreas	A/duck meat/Anyang/01(H5N1)		Brown et al., 2008b
Charadriiformes	Laughing gull^b^ (*Leucophaeus atricilla)*	E	Brain, Pancreas, Adrenal glands, Heart (minimal), Lungs (minimal), Air sacs (minimal), Thymus (minimal), Kidneys (minimal), Small intestines (minimal) and Eyes (minimal)	A/Whooper Swan/Mongolia/244/05 (H5N1) and A/Duck Meat/Anyang/01 (H5N1)	Viral antigen frequently detected in endothelial cells in brain	Brown et al., 2006
Charadriiformes	Laughing gulls (*Larus atricilla*)	E	None	A/chicken/Hong Kong/220/97 (chicken/HK) (H5N1)		Perkins and Swayne, 2003
Charadriiformes	Laughing gulls (*Larus atricilla*)	E	None	A/chicken/ Hong Kong/220/97 (H5N1)		Perkins and Swayne, 2002b

a E, Experimental; N, Natural.

b Viral antigen recorded in birds that died during the experiment.

## Humans

**Figure 2:**
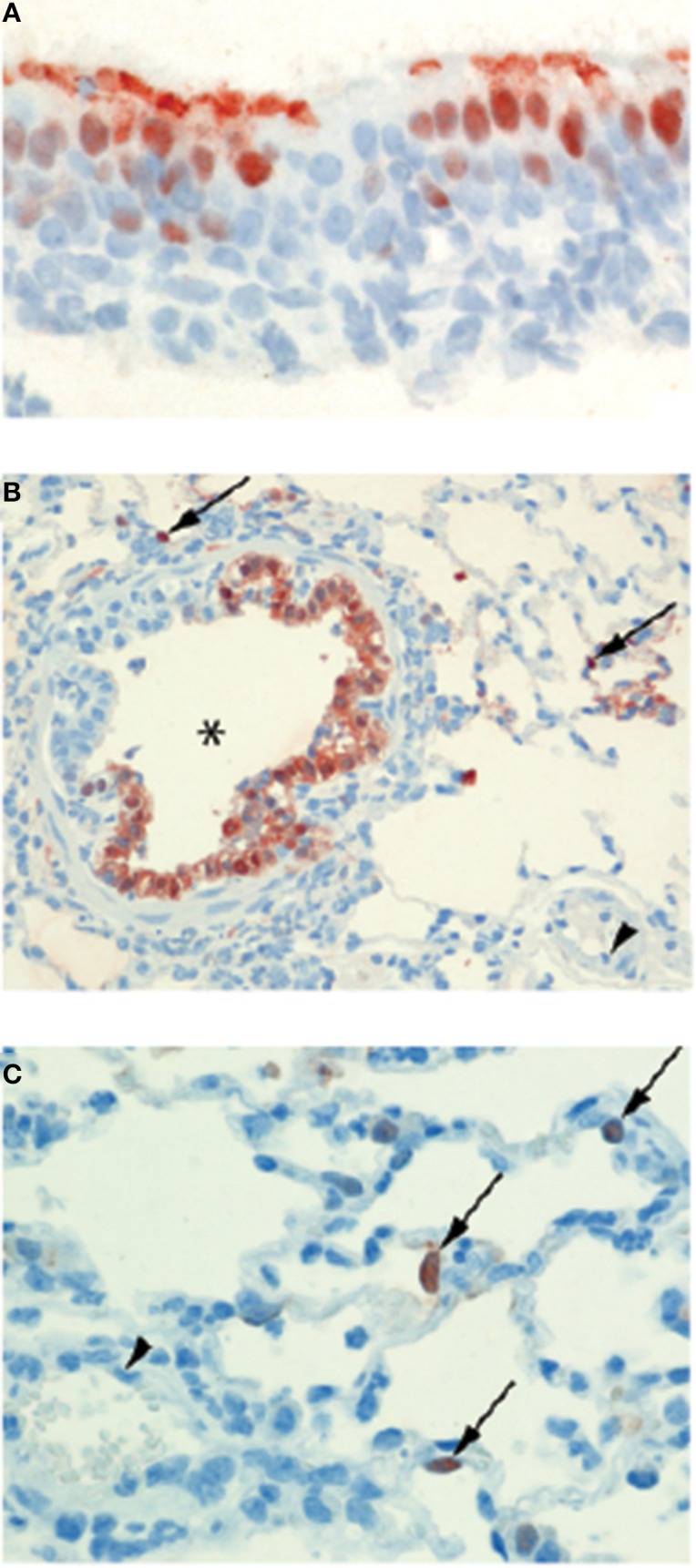
**Epitheliotropism of IAVs in ferrets**. IAV distribution in: **(A)** Epithelial cell nuclei and apical cytoplasm of the nasal respiratory mucosa of a ferret 24 h after experimental intranasal infection with 10^6^ TCID_50_ of seasonal influenza H3N2 A/Netherlands/177/2008 (original magnification 800×). **(B)** Epithelial cells of a bronchiole (asterisk) and in few alveolar lining epithelial cell nuclei (arrows) of a ferret lung 24 h after experimental intratracheal infection with 10^6^ TCID_50_ of influenza pH1N1 A/Netherlands/602/2009. The blood vessel lumen lining endothelial cell nuclei (arrowhead) do not stain positive for viral antigen (original magnification 200×). **(C)** Alveolar lining epithelial cell nuclei (arrows) of a ferret lung 24 h after experimental intratracheal infection with 10^6^ TCID_50_ of HPAI H5N1 virus A/Indonesia/05/2005. The blood vessel lumen lining endothelial cell nuclei (arrowhead) do not stain positive for viral antigen (original magnification 400×). Brown-reddish staining antigen indicative of viral replication was present only in epithelial cells of ferret respiratory tract, not in ferret endothelial cells. (Immunohistochemistry (IHC) for IAV-nucleoprotein (NP) with hematoxylin counterstain).

In humans, the primary cellular targets of IAV are epithelial cells in the respiratory tract. Seasonal IAVs, which are adapted to and circulate in the human population, typically infect ciliated cells in the upper respiratory tract, trachea and bronchi (van Riel et al., 2007b, 2010). In contrast, HPAI viruses such as H5N1 preferentially infect the lower respiratory tract, specifically club cells and alveolar type II pneumocytes (van Riel et al., 2006, 2007a). This differential tropism reflects, in part, the ability of H5N1 viruses to bind to α-2,3-linked sialosaccharides (expressed on type II pneumocytes) whilst seasonal IAVs typically display a preference for α-2,6-linked sialic acid (Shinya et al., 2006; van Riel et al., 2006, 2007a). Within the lower respiratory tract, alveolar epithelial cells are in close proximity to the underlying endothelium. Indeed, in the human alveolus there is on average only 0.5 μm separating the airspace from the capillary (Piantadosi and Schwartz, 2004). During IAV infection the endothelium is therefore likely to be exposed to free virus particles produced by infected and damaged epithelial cells. It is often suggested that—like chickens and black swans—IAV infects human endothelial cells, and that this contributes to disease pathogenesis (Chan et al., 2009; Ocaña-Macchi et al., 2009; Armstrong et al., 2012, 2013; Zeng et al., 2012). For example, *in vitro* studies using primary human lung microvascular endothelial cells demonstrated that endothelial cells can be infected by seasonal H3N2 IAV, with infection ultimately resulting in increased endothelial cell permeability (Armstrong et al., 2012). Others have suggested that IAV infection of the pulmonary endothelium is a unique feature of infection with H5N1 viruses, as H5N1 strains are able to efficiently infect and replicate in human microvascular endothelial cells whereas other IAV strains do not (Chan et al., 2009; Ocaña-Macchi et al., 2009; Zeng et al., 2012). However, in spite of these *in vitro* studies, there is limited evidence suggesting that IAV infection of human endothelial cells occurs *in vivo*. Post mortem analysis of patients who succumbed to H5N1 did not demonstrate the presence of virus in pulmonary endothelial cells (Gu et al., 2007). Similarly, endothelial cells were only very infrequently infected in a limited number of patients infected with fatal pandemic 2009 H1N1 (Shieh et al., 2010). Whilst one recent study in mice recorded endothelial cell infection (Ogiwara et al., 2014), in most animal models of human infection IAV infection of the endothelium is rarely observed (Kuiken et al., 2010) (see Figure 2). Together, these data suggest that infection of endothelial cells by IAV is unlikely to contribute to disease severity in humans.

Although human endothelial cells are not infected by IAV *in vivo*, endothelial cells may still play an important role in the pathogenesis of IAV in humans. During IAV infection pulmonary endothelial cells are thought to be the most important source of cytokines in the lung (Teijaro et al., 2011). Specifically, in a mouse model of influenza, treatment with a SIP1 receptor agonist reduced IAV-induced mortality by blocking endothelial cell cytokine and chemokine production (Teijaro et al., 2011), suggesting a key role for endothelial cells in IAV pathogenesis. It has also been shown that the IAV-induced inflammatory response (namely the production of TNFα, IL-6 and IL-1β) upregulates trypsin production (Wang et al., 2010). The increased amount of trypsin then damages the tight junction protein zona-occludens 1 that is found between endothelial cells and increases endothelial permeability (Wang et al., 2010). However, it is important to note that this is unlikely to account for the pulmonary oedema observed during severe IAV infection as it is epithelial, not endothelial, cells that play the most important role in ensuring that the alveolus remains free of fluid (Gorin and Stewart, 1979). Pro-inflammatory cytokines, derived either from the endothelium or other cells in the lung, may also contribute to the development of thrombosis during IAV infection (Babinska et al., 2002; Armstrong et al., 2013). For example, treatment of human umbilical vein endothelial cells with TNFα significantly increased platelet binding to the cells by promoting the interaction between the F11 receptor on platelets and the F11 receptor on endothelial cells (Babinska et al., 2002). This observation is supported by epidemiological evidence from the 2009 H1N1 pandemic, whereby 5.9% of patients hospitalized for influenza virus infection had thrombotic vascular events (Bunce et al., 2011).

**Endothelial cell infection in cats.**

Upon the initial emergence of H5N1 viruses, mortality in cats was observed in areas where the viruses were spreading in wild and domestic birds. This suggested that cats were susceptible to infection. This was unusual as cats have long been considered to be refractory to IAV infection. H5N1 viruses administered to cats intratracheally resulted in productive infection of many organs, including the respiratory tract, with parenchymal and epithelial cells as the primary targets for viral replication (Rimmelzwaan et al., 2006). These studies demonstrated that domestic cats could indeed develop clinical disease upon H5N1 infection. Cats and other carnivores can be exposed to H5N1 viruses by feeding on sick or dead birds. In order to mimic this route of infection, Reperant and colleagues (2012) administered H5N1 to the small intestine of cats using enteric-coated capsules (the use of which avoided accidently exposing the respiratory tract to the inoculum). Three days post-infection H5N1 infected cats became lethargic and began to display severe clinical signs. Surprisingly, immunohistochemistry demonstrated that there was an overwhelming infection of endothelial cells in virtually every organ of infected cats, in a pattern reminiscent of that observed in chickens. In contrast, parenchymal cells were rarely infected. In particular, infection of respiratory epithelial cells was not observed, despite massive infection of the pulmonary endothelium. The virus used to infect the cats via the intestine had been isolated from the liver of infected chickens, and may have accumulated mutations potentially responsible for such difference in tissue tropism. However, analyses of the viruses used to infect, and recovered from, cats inoculated intra-tracheally and via the intestine, revealed no coding differences associated with the difference in tropism. These data suggest that the route of virus exposure may influence the role of the endothelium in the pathogenesis of influenza virus in mammals.

In addition to mediating cytokine production, endothelial cells may also indirectly control the inflammatory response in the lung during IAV infection via the expression of adhesion molecules, such as E-selectin, P-selectin, ICAM1, and VCAM1, on their apical surface. These adhesion molecules can bind to various leukocytes and mediate their extravasation to the infected lung. The increased expression of E/P-selectin expression on human endothelial cells following exposure to H5N1 (Perrone et al., 2008) may therefore account for the increased inflammatory response (and lung lesions) associated with this virus. In sum, whilst human endothelial cells are not infected with IAV, endothelial cells still play an important role in the pathogenesis of IAV in humans.

## Conclusions and future directions

Endothelial cells play important but distinct roles in the pathogenesis of IAV in wild birds, poultry and humans. Whilst endothelial cells are infected by HPAI viruses in chickens and swans, in humans they are more important in driving and controlling the inflammatory response in the lung. It is important to note that endothelial cells in both chickens and swans may also influence the inflammatory response to IAV. It has already been suggested that the overwhelming endothelial tropism of H5N1 viruses in poultry may disrupt the innate immune response (Suzuki et al., 2009). However, the details of this ‘disruption’ have been hard to elucidate due to the limited availability of reagents to study the avian immune response. This remains a key research priority for the future. In addition, what makes the endothelial cells of chickens and black swans (and not those of other wild bird species and humans) so permissive to H5N1 viruses *in vivo* remains to be determined. It is likely that as research and the availability of reagents for studying avian species continues to grow new roles for endothelial cells in pathogenesis of IAV will be discovered. However, what is clear at present is that endothelial cells contribute to the severity of IAV infections across multiple different species.

### Conflict of interest statement

The authors declare that the research was conducted in the absence of any commercial or financial relationships that could be construed as a potential conflict of interest.
